# Physical inactivity and sedentary behaviors in the Bangladeshi population during the COVID-19 pandemic: An online cross-sectional survey

**DOI:** 10.1016/j.heliyon.2020.e05392

**Published:** 2020-10-30

**Authors:** Md. Estiar Rahman, Md. Saiful Islam, Md. Sajan Bishwas, Mst. Sabrina Moonajilin, David Gozal

**Affiliations:** a Department of Public Health and Informatics, Jahangirnagar University, Savar, Dhaka 1342, Bangladesh; b Youth Research Association, Savar, Dhaka 1342, Bangladesh; c Department of Child Health and the Child Health Research Institute, The University of Missouri School of Medicine, Columbia, MO 65201, USA

**Keywords:** Public health, Quality of life, Epidemiology, Physical activity, Health education, Physical exercise, Categories, Leisure, Quarantine, Lockdown

## Abstract

This study aimed to determine the prevalence of physical inactivity and sedentary behaviors during the COVID-19 pandemic among Bangladeshi people. An online survey was conducted among 2,028 people over a period of 10 days on June, 2020 during the COVID-19 pandemic at a time that the number of newly diagnosed cases was increasing, lockdown was still in place. Survey questions included socio-demographics and an adapted version of the IPAQ-SF to assess physical activity and sedentary behaviors. The prevalence rates of physical inactivity (<600 MET–minutes/week) and high sedentary behaviors (≥8 h/day) among Bangladeshi people were 37.9% and 20.9%, respectively. Regression analyses revealed that young age, being a student, from a middle-class family, or upper-class family, living with nuclear family, urban living, and suffering from no chronic diseases were all associated with physical inactivity and high sedentary behaviors. Moreover, physical inactivity and high sedentary behavior were strongly interrelated. However, many of the univariate risk factors exhibited interdependency. During the COVID-19 pandemic coinciding with lockdown measures a sizeable proportion of Bangladeshi people were physically inactive and reported sedentary behaviors ≥8 h/day. Public campaigns and media-based interventions encouraging home-based physical activities should be promoted to attenuate the impact of lockdown measures during a pandemic.

## 1 Introduction

The outbreak of coronavirus disease 2019 (COVID-19) caused by Severe Acute Respiratory Syndrome Coronavirus 2 (SARS-CoV-2), has become a global public health threat [1, 2]. The outbreak was first revealed in Wuhan city, in the Hubei Province of China, in late December 2019 [3]. Since then, the virus has spread worldwide, with millions of COVID-19 cases and related deaths being recorded globally [4]. The first case of COVID-19 was confirmed in Bangladesh on March 8, 2020 [5, 6, 7], and more than 317,500 confirmed cases of COVID-19 and 4,351 deaths have been recorded by September 03, 2020 [8].

Physical activity (PA) is regarded as a critical component of a healthy lifestyle and disease prevention [9]. Conversely, physical inactivity increases the risk of many chronic diseases, such as hypertension, coronary heart disease, stroke, diabetes, depression, and risk of falls [10]. Regular physical activity helps maintain a healthy weight, reduces the risk of developing obesity, and strengthens the immune system [11, 12]. Physical activity also reduces feelings of depression and improves mental health [13]. Considering the health benefits of regular physical activity, the WHO recommends that individuals aged 18–64 years should engage in >150 min of moderate-intensity or >75 min of vigorous-intensity physical activity per week or an equivalent combination of moderate- and vigorous-intensity activity [14]. Sedentary behavior refers to any waking behavior characterized by an energy expenditure not exceeding 1.5 metabolic equivalents (METs), while in a sitting or reclining position [15, 16].

Like the vast majority of the affected countries around the world, Bangladesh initiated a lock-down policy to ensure spatial distancing, self-isolation, or quarantine, as part of the efforts to limit the spread of COVID-19. The government declared a nationwide lockdown from March 26 to May 30, with a 7^th^ extension being recently announced [17, 18]. Previous studies investigated the impact of COVID-19 on physical activity in different age clusters and in different regions [19, 20, 21]. Reports have indicated that the COVID-19 pandemic-related public health restrictions appear to have led to reductions in physical activity [22, 23, 24, 25]. We hypothesized that the extended periods of lockdown in Bangladesh may have adversely impacted physical activity and increased sedentary behaviors in the population. We aimed to determine the prevalence of physical inactivity and sedentary behaviors during the COVID-19 pandemic among Bangladeshi people.

## 2 Materials and methods

### 2.1 Study design

We adopted an online cross-sectional survey approach to assess the levels of physical activity and sedentary behaviors among Bangladeshi citizens during the COVID-19 pandemic. The survey was carried out between June 20 to June 30, 2020, when the number of newly diagnosed cases increasing, and the government continued to impose lockdown restrictions to limit the spread of COVID-19. The target population was the general Bangladeshi population. Inclusion criteria were being (i) a Bangladeshi residence, (ii) aged 18 years or older, and (iii) being able to read Bangla.

### 2.2 Study procedures

Participants were recruited from various social media platforms (e.g., Facebook, WhatsApp), using convenience sampling. Data were collected by means of an anonymous online questionnaire. The questionnaire was translated into Bangla (the native language of participants), and then back-translated to English by different experts to assess validity. The most widely used standardized procedure (i.e., Beaton et al., 2000) was used to perform the back translation for this questionnaire [26], which has been previously used in Bangladesh [27, 28]. A pilot test was conducted on 50 samples to test the validity of the questionnaire. The data from the pilot survey were not included in the final analysis. The online survey was conducted using a survey link created on Google Form. A total of 2,083 people completed the online survey. Of these, 55 were excluded as they were below 18 years of age. Therefore, the final sample consisted of 2,028 participants.

### 2.3 Measures

#### 2.3.1 Socio-demographic measures

Socio-demographic variables included in the survey were age (later categorized: young [18–25 years], and adult [25 + years]), gender (male vs. female), marital status (unmarried, married, and divorced/widows/widowers), education levels (secondary/below, higher secondary, and graduation/above), occupation (student, housewife, employed, businessman, and unemployed), monthly family income (later categorized: lower-class [<15,000 Bangladeshi Taka (BDT)], middle-class [15,000–30,000 BDT], and upper-class [>30,000 BDT]), family type (nuclear vs. joint), and current place of residence (village, sub-district town, district town, and divisional town). Other variables included were self-reported physical health (good, moderate, and poor), chronic diseases (yes vs. no), and cigarette smoking currently (yes vs. no).

#### 2.3.2 Assessment of physical activity level and sedentary behavior

Physical activity level was assessed using the International Physical Activity Questionnaire Short Form (IPAQ-SF) [29]. The IPAQ-SF is a valid and reliable tool for physical activity surveillance across a range of populations [30, 31, 32, 33, 34]. Validity of the IPAQ-SF has been assessed across 12 countries, and showed that acceptable properties for use in many settings and in different languages [35]. The IPAQ-SF consists of 6 items providing information on time spent in walking, moderate- and vigorous-intensity activities during a typical week. Walking activities are defined as “walking at home and at work, walking to travel from place to place, and any other walking done solely for recreation, sport, exercise, or leisure”. Moderate-intensity activities are defined as “those that take moderate physical effort and produce a moderate increase in respiration rate”, and included examples such as carrying light objects, working in the garden, cycling at a regular pace, or doing prolonged physical work at home. Vigorous physical activities are defined as “those that take hard physical effort and produce vigorous increases in respiration rate’ such as lifting heavy objects, hoeing the earth, practicing zumba, cycling on an exercise bike, or running on a treadmill at high speed” [29]. For each of walking, moderate- and vigorous-intensity activities, individuals were asked to report the number of days per week that they performed the targeted activity for at least 10 min at a time, and then asked to report how much time they usually spent on one of those days doing the targeted physical activity.

According to the IPAQ data analysis guideline, all activity data were converted to Metabolic Equivalent Task (MET), the standard unit used to express the intensity of physical activities. For all three types of activities (i.e., walking, moderate- and vigorous-intensity activities), MET-minutes per week were calculated as follows: walking = (3.3 × walking min × walking days); moderate activity = (4.0 × moderate activity min × moderate activity days); vigorous activity = (8.0 × vigorous activity min × vigorous activity days). Physical activity levels for each participant were classified into 3 categories based on the MET–minutes/week of the total weekly energy expenditure (i.e., the sum of walking, moderate- and vigorous-intensity physical activities): (i) low (<600 MET–minutes/week); (ii) moderate active (≥600 MET–minutes/week); (iii) high active (≥3000 MET–minutes/week) [29]. For ease of regression analysis, physical activity level was later categorized: inactive (low active) and active (moderate/high active).

The IPAQ-SF has an additional item, namely “During the last week, how long in total did you spend in sedentary activities on a typical day?” to assess sedentary behavior. Sedentary activities were defined as “those activities in a sitting, reclining, or lying position (except sleep) requiring very low energy expenditure” and examples included sitting/lying down reading or watching TV, computer use, video games, etc. Sedentary behaviors for each participant were categorized as: <8 h/day and ≥8 h/day (high), as used in previous studies [36, 37]. This categorization is based on a previous cohort study that reported a detrimental association between SB ≥ 8 h/day and all-cause mortality [38].

### 2.4 Statistical analysis

Statistical analysis was performed using Microsoft Excel 2019 and Statistical Package for Social Science (SPSS) version 25 (Chicago, IL). Microsoft Excel was used for data entry, editing, and sorting. Continuous data were presented as mean and standard deviation (SD), and categorical data as frequency and percentage. The chi-square test was applied for categorical variables. Logistic regression (both unadjusted and adjusted models) was performed with a 95% confidence interval to determine the significant associations between categorical dependent and independent variables. Analyses were univariate, yielding crude odds ratios, followed by multivariable analyses with predictors combined, with the exception of sedentary behavior and physical inactivity in the models of each other, and yielding adjusted odds ratios. The association of variables was considered statistically significant if the two-sided *p*-value was less than 0.05.

### 2.5 Ethics

The present study was carried out in accordance with the guidelines of the Helsinki Declaration, 1975. In addition, the formal ethics approval was granted by the Ethical Review Committee, the Faculty of Biological Sciences, Jahangirnagar University, Savar, Dhaka-1342, Bangladesh (Ref No: BBEC, JU/M 2020 (7)1). Participants were well informed about the procedure and purpose of the study, and confidentiality of their information. Informed consent was ensured by each of participants. Furthermore, all data were collected anonymously and analyzed by using the coding system.

## 3 Results

### 3.1 General characteristics of participants

A total of 2,028 participants were included in the final analysis. Of these, 57.2% were male, the mean age was 25.9 years (SD = 8.1) and age ranged 18–65 years. The majority were single (71.1%), had graduation or above level education (64.4%), and were students (60.9%). A sizeable majority were from middle-class families (41.5%), came from nuclear families (69.9%), and were from village areas (32.5%). Good physical health reported in 53.4% of responders, with nearly one-fifth of participants indicating chronic diseases (18.3%), and the majority (78.4%) did not smoke currently (Table 1).

**Table 1:** Distribution of variables, and association with physical inactivity and sedentary behaviors (N = 2,028).

Variables	Total N = 2028	Physical inactivity (n = 768; 37.9%)				Sedentary behavior (n = 424; 20.9%)			
	n (%)	n (%)	χ^2^	*df*	*p*-value	n (%)	χ^2^	*df*	*p*-value
**Age**									
Young (18–25 years)	1389 (68.5)	591 (42.5)	41.015	1	<0.001	341 (24.6)	35.375	1	<0.001
Adult (>25 years)	639 (31.5)	177 (27.7)				83 (13.0)			

**Gender**									
Male	1161 (57.2)	452 (38.9)	1.302	1	0.254	208 (17.9)	14.699	1	<0.001
Female	867 (42.8)	316 (36.4)				216 (24.9)			

**Marital status**									
Single	1442 (71.1)	639 (44.3)	88.119	2	<0.001	360 (25.0)	51.273	2	<0.001
Married	556 (27.4)	123 (22.1)				58 (10.4)			
Divorced/widows/widowers	30 (1.5)	6 (20.0)				6 (20.0)			

**Education level**									
Secondary/below	223 (11.0)	44 (19.7)	57.985	2	<0.001	43 (19.3)	1.562	2	0.458
Higher secondary	499 (24.6)	156 (31.3)				97 (19.4)			
Graduation/above	1306 (64.4)	568 (43.5)				284 (21.7)			

**Occupation**									
Student	1236 (60.9)	569 (46.0)	105.702	4	<0.001	318 (25.7)	44.912	4	<0.001
Housewife	175 (8.6)	23 (13.1)				24 (13.7)			
Employed	360 (17.8)	95 (26.4)				51 (14.2)			
Businessman	171 (8.4)	51 (29.8)				21 (12.3)			
Unemployed	86 (4.2)	30 (34.9)				10 (11.6)			

**Monthly family income**									
Lower class	396 (19.5)	91 (23.0)	69.685	2	<0.001	59 (14.9)	18.131	2	<0.001
Middle class	842 (41.5)	302 (35.9)				166 (19.7)			
Upper class	790 (39.0)	375 (47.5)				199 (25.2)			

**Family type**									
Nuclear	1418 (69.9)	595 (42.0)	33.528	1	<0.001	323 (22.8)	9.983	1	0.002
Join	610 (30.1)	173 (28.4)				101 (16.6)			

**Current place of residence**									
Village	659 (32.5)	217 (32.9)	72.154	3	<0.001	105 (15.9)	95.843	3	<0.001
Sub-district town	449 (22.1)	130 (29.0)				57 (12.7)			
District town	373 (18.4)	134 (35.9)				70 (18.8)			
Division town∗	547 (27.0)	287 (52.5)				192 (35.1)			

**Self-reported physical health**									
Good	1082 (53.4)	388 (35.9)	10.308	2	0.006	250 (23.1)	12.949	2	0.002
Moderate	805 (39.7)	310 (38.5)				137 (17.0)			
Poor	141 (7.0)	70 (49.6)				37 (26.2)			

**Chronic diseases**									
Yes	371 (18.3)	106 (28.6)	16.686	1	<0.001	61 (16.4)	5.475	1	0.019
No	1657 (81.7)	662 (40.0)				363 (21.9)			

**Smoking currently**									
Yes	438 (21.6)	161 (36.8)	0.294	1	0.588	79 (18.0)	2.784	1	0.095
No	1590 (78.4)	607 (38.2)				345 (21.7)			

**Physical activity level**									
Moderate/high	1260 (62.1)	0 (0.0)	―	―	―	183 (14.5)	81.990	1	<0.001
Inactive	768 (37.9)	768 (100.0)				241 (31.4)			

**Sedentary behavior**									
No	1604 (79.1)	527 (32.9)	81.990	1	<0.001	0 (0.0)	―	―	―
Yes	424 (20.9)	241 (56.8)				424 (100.0)			

∗ Division town – urban densely populated setting.

### 3.2 Physical inactivity and sedentary behavior

**Figure 1:**
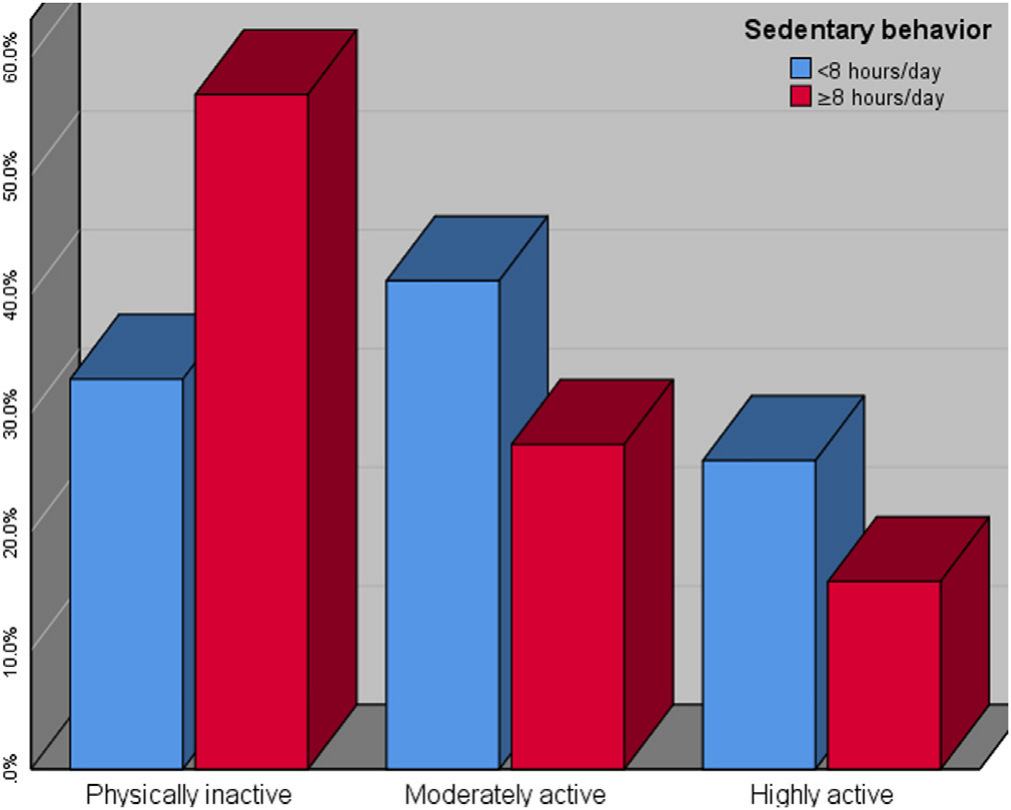
Interrelation between physical activity level and sedentary behaviors among Bangladeshi people during the COVID-19 pandemic.

Analysis of the IPAQ-SF scale demonstrated that 37.9% participants were physically inactive during the COVID-19 pandemic. In addition, 38.3% participants were moderately active, and 23.9% had high levels of physical activity. Regarding sedentary behaviors, 20.9% participants reported high sedentary behavior (≥8 h/day). Figure 1 represents the interrelation between physical activity level and sedentary behaviors among Bangladeshi people during the COVID-19 pandemic.

### 3.3 Categorical comparisons of physical inactivity and sedentary behavior

The proportion of physical inactivity was significantly higher among (i) young (18–25 years) vs. adults (>25 years) (42.5% vs. 27.7%, *p* < 0.001), (ii) single vs. other (44.3% vs. 20.0%, *p* < 0.001) (iii) participants with graduation/above vs. secondary (6–10 grades)/below education level (43.5% vs. 19.7%, *p* < 0.001), (iv) student vs. housewife (46.0% vs. 13.1%, *p* < 0.001), (v) participants from upper-class vs. lower-class family (47.5% vs. 23.0%, *p* < 0.001), (ⅵ) participants those living with nuclear vs. join family (42.0% vs. 28.4%, *p* < 0.001), (ⅶ) participants those living in divisional town vs. sub-district town (52.5% vs. 29.0%, *p* < 0.001), (ⅷ) participants with poor vs. good physical health (49.6% vs. 35.9%, *p* = 0.006), (ⅸ) participants without chronic diseases vs. those who had chronic diseases (40.0% vs. 28.6%, *p* < 0.001), and (ⅹ) participants with vs. without high sedentary behavior (56.8% vs. 32.9%, *p* < 0.001) (Table 1).

The proportion of sedentary behavior was significantly higher among (i) young vs. adult (24.6% vs. 13.0%, *p* < 0.001), (ii) female vs. male (24.9% vs. 17.9%, *p* < 0.001), (iii) single vs. married participants (25.0% vs. 10.4%, *p* < 0.001), (iv) students vs. unemployed participants (25.7% vs. 11.6%, *p* < 0.001), (v) participants from upper class vs. lower class family (25.2% vs. 14.9%, *p* < 0.001), (ⅵ) participants those living with nuclear vs. join family (22.8% vs. 16.6%, *p* = 0.002), (ⅶ) participants those living in division vs. sub-district town (35.1% vs. 12.7%, *p* < 0.001), (ⅷ) participants without chronic diseases vs. those who had (21.9% vs. 16.4%, *p* = 0.019), (ⅸ) participants with poor vs. good physical health (26.2% vs. 17.0%, *p* = 0.002), and (ⅹ) participants with vs. without considerable physical inactivity (31.4% vs. 14.5%, *p* < 0.001) (Table 1).

### 3.4 Logistic regression analysis of physical inactivity and sedentary behaviors

Young people were 1.9 times more likely to be physically inactive (OR_PI_ = 1.9; 95% CI = 1.6–2.4, *p* < 0.001), and 2.2 times more likely to develop high sedentary behaviors (OR_SB_ = 2.2; 95% CI = 1.7–2.8, *p* < 0.001) compared to adults (Table 2). Other predictors of physical inactivity (PI) and high sedentary behavior (SB) were being students (OR_PI_ = 1.6; 95% CI = 1.0–2.5, *p* = 0.046, and OR_SB_ = 2.6; 95% CI = 1.3–5.1, *p* = 0.005), being from middle class families (OR_PI_ = 1.9; 95% CI = 1.4–2.5, *p* < 0.001, and OR_SB_ = 1.4; 95% CI = 1.0–1.9, *p* = 0.041), being from upper-class families (OR_PI_ = 3.0; 95% CI = 2.3–4.0, *p* < 0.001, and OR_SB_ = 1.9; 95% CI = 1.4–2.6, *p* < 0.001), living with nuclear families (OR_PI_ = 1.8; 95% CI = 1.5–2.2, *p* < 0.001, and OR_SB_ = 1.5; 95% CI = 1.2–1.9, *p* = 0.002), living in urban environment (OR_PI_ = 2.2; 95% CI = 1.8–2.8, *p* < .001, and OR_SB_ = 2.9; 95% CI = 2.2–3.7, *p* < .001), and not suffering from chronic diseases (OR_PI_ = 1.7; 95% CI = 1.3–2.1, *p* < .001, and OR_SB_ = 1.4; 95% CI = 1.1–1.9, *p* = 0.02) (Table 2). Additional risk factors of physical inactivity were being single (OR_PI_ = 3.2; 95% CI = 1.3–7.8, *p* = 0.012), having higher secondary level education (OR_PI_ = 1.9; 95% CI = 1.3–2.7, *p* = 0.002), having graduation/above level education (OR_PI_ = 3.1; 95% CI = 2.2–4.4, *p* < 0.001), having poor physical health (OR_PI_ = 1.8; 95% CI = 1.2–2.5, *p* = 0.002); in contrast, being housewife was protective (OR_PI_ = 0.3; 95% CI = 0.2–0.5, *p* < 0.001). Likewise, additional risk factors of high sedentary behaviors were being female (OR_SB_ = 1.5; 95% CI = 1.2–1.9, *p* < 0.001); conversely, moderate physical condition was a protective factor (OR_SB_ = 0.7; 95% CI = 0.5–0.9, *p* = 0.001) (Table 2).

**Table 2:** Regression analysis (univariate analysis) by physical inactivity and sedentary behavior.

Variables	Physical inactivity					Sedentary behavior				
	B	S.E.	Exp(B)	95% CI for Exp(B)	*p*-value	B	S.E.	Exp(B)	95% CI for Exp(B)	*p*-value
**Age (ref. adult)**										
Young (18–25 years)	0.659	0.104	1.933	(1.577–2.369)	<0.001	0.779	0.133	2.180	(1.679–2.830)	<0.001

**Gender (ref. male)**										
Female	-0.106	0.093	0.900	(0.750–1.079)	0.254	0.419	0.110	1.520	(1.226–1.885)	<0.001

**Marital status (ref. divorced, widows, or widowers)**										
Single	1.158	0.460	3.183	(1.293–7.834)	0.012	0.286	0.460	1.331	(0.540–3.282)	0.535
Married	0.128	0.468	1.136	(0.454–2.842)	0.785	-0.764	0.477	0.466	(0.183–1.187)	0.109

**Education level (ref. secondary or below)**										
Higher secondary	0.615	0.194	1.850	(1.265–2.706)	0.002	0.010	0.204	1.010	(0.677–1.507)	0.961
Graduation or above	1.141	0.177	3.131	(2.212–4.432)	<0.001	0.151	0.183	1.163	(0.813–1.664)	0.407

**Occupation (ref. unemployed)**										
Student	0.465	0.233	1.592	(1.008–2.516)	0.046	0.968	0.343	2.633	(1.345–5.153)	0.005
Housewife	-1.264	0.318	0.282	(0.151–0.527)	<0.001	0.189	0.402	1.208	(0.550–2.655)	0.638
Employed	-0.402	0.256	0.669	(0.405–1.105)	0.116	0.227	0.369	1.254	(0.609–2.584)	0.539
Businessman	-0.232	0.281	0.793	(0.457–1.377)	0.411	0.062	0.409	1.064	(0.477–2.373)	0.880

**Monthly family income (ref. lower class)**										
Middle class	0.628	0.139	1.874	(1.426–2.463)	<0.001	0.338	0.166	1.403	(1.014–1.940)	0.041
Upper class	1.108	0.139	3.029	(2.306–3.978)	<0.001	0.654	0.163	1.923	(1.397–2.648)	<0.001

**Family type (ref. join)**										
Nuclear	0.602	0.105	1.826	(1.487–2.242)	<0.001	0.396	0.126	1.487	(1.161–1.903)	0.002

**Current place of residence (ref. village)**										
Sub-district town	-0.186	0.133	0.830	(0.640–1.077)	0.162	-0.265	0.177	0.767	(0.542–1.086)	0.135
District town	0.133	0.136	1.142	(0.875–1.491)	0.329	0.198	0.170	1.219	(0.873–1.701)	0.244
Division town	0.810	0.119	2.248	(1.780–2.840)	<0.001	1.049	0.139	2.854	(2.173–3.748)	<0.001

**Self-reported physical health (ref. good)**										
Moderate	0.113	0.096	1.120	(0.928–1.353)	0.238	-0.382	0.118	0.683	(0.541–0.861)	0.001
Poor	0.567	0.180	1.763	(1.239–2.509)	0.002	0.169	0.205	1.184	(0.793–1.768)	0.409

**Chronic diseases (ref. yes)**										
No	0.509	0.125	1.663	(1.301–2.127)	<0.001	0.355	0.152	1.426	(1.058–1.921)	0.020

**Smoking currently (ref. no)**										
Yes	-0.061	0.112	0.941	(0.756–1.172)	0.588	-0.231	0.138	0.794	(0.605–1.042)	0.096

**Physical activity level (ref. moderate/high)**										
Inactive	―	―	―	―	―	0.990	0.112	2.691	(2.163–3.349)	<0.001

**Sedentary behavior (ref. no)**										
Yes	0.990	0.112	2.691	(2.163–3.349)	<0.001	―	―	―	―	―

B=Regression coefficient; S.E. = Standard Error; Exp(B) = Odds Ratio (OR); CI = Confidence Interval.

Furthermore, the findings showed a significant association between physical inactivity and high sedentary behaviors (χ^2^ = 82.0; df = 1, *p* < 0.001), as well as physical inactivity (OR_SB_ = 2.7; 95% CI = 2.2–3.3, *p* < 0.001) and sedentary behaviors (OR_PI_ = 2.7; 95% CI = 12.2–3.3, *p* < 0.001) emerged as significant predictors of each other (Table 2).

Multivariable logistic regression analyses with each of the factors entered into the model revealed that most of the risk factors were inter-dependent and lost statistical significance (Table 3). For physical inactivity, significant associations with education level, residence, self-reported physical health, and sedentary behavior emerged. For sedentary behaviors, the only significant associations retained in the model included gender, education level, self-reported physical health, and physical inactivity (Table 3).

**Table 3:** Regression analysis (multivariate analysis) as related to physical inactivity and sedentary behaviors.

Variables	Physical inactivity^a^					Sedentary behavior^b^				
	B	S.E.	Exp(B)	95% CI for Exp(B)	*p*-value	B	S.E.	Exp(B)	95% CI for Exp(B)	*p*-value
**Age (ref. adult)**										
Young (18–25 years)	-0.148	0.171	0.863	(0.617–1.207)	0.515	0.132	0.203	1.142	(0.767–1.700)	0.515

**Gender (ref. male)**										
Female	0.277	0.117	1.319	(1.049–1.658)	0.028	-0.294	0.133	0.746	(0.574–0.968)	0.028

**Marital status (ref. divorced, widows, or widowers)**										
Single	0.598	0.509	1.819	(0.670–4.936)	0.812	0.120	0.506	1.128	(0.419–3.037)	0.812
Married	0.212	0.506	1.236	(0.458–3.330)	0.170	-0.694	0.506	0.500	(0.185–1.347)	0.170

**Education level (ref. secondary or below)**										
Higher secondary	0.354	0.215	1.425	(0.934–2.172)	0.113	-0.367	0.232	0.692	(0.440–1.091)	0.113
Graduation or above	0.566	0.209	1.761	(1.170–2.652)	0.006	-0.625	0.228	0.535	(0.342–0.837)	0.006

**Occupation (ref. unemployed)**										
Student	0.193	0.272	1.213	(0.712–2.067)	0.109	0.596	0.372	1.815	(0.876–3.762)	0.109
Housewife	-0.828	0.358	0.437	(0.217–0.881)	0.189	0.590	0.449	1.803	(0.749–4.344)	0.189
Employed	-0.463	0.281	0.630	(0.363–1.092)	0.110	0.624	0.390	1.866	(0.869–4.009)	0.110
Businessman	-0.125	0.307	0.882	(0.484–1.609)	0.439	0.334	0.432	1.397	(0.599–3.257)	0.439

**Monthly family income (ref. lower class)**										
Middle class	0.692	0.149	1.999	(1.492–2.677)	0.123	0.270	0.175	1.311	(0.929–1.848)	0.123
Upper class	0.951	0.160	2.589	(1.891–3.543)	0.197	0.246	0.191	1.279	(0.880–1.859)	0.197

**Family type (ref. join)**										
Nuclear	0.390	0.117	1.477	(1.175–1.856)	0.961	0.007	0.140	1.007	(0.765–1.325)	0.961

**Current place of residence (ref. village)**										
Sub-district town	-0.285	0.146	0.752	(0.566–1.000)	<0.001	-0.834	0.166	0.434	(0.314–0.601)	<0.001
District town	-0.582	0.153	0.559	(0.414–0.755)	<0.001	-1.069	0.184	0.343	(0.239–0.493)	<0.001
Division town	-0.416	0.152	0.659	(0.490–0.888)	<0.001	-0.712	0.172	0.490	(0.350–0.687)	<0.001

**Self-reported physical health (ref. good)**										
Moderate	0.385	0.109	1.469	(1.187–1.818)	0.002	-0.403	0.130	0.668	(0.518–0.861)	0.002
Poor	0.980	0.204	2.666	(1.787–3.977)	0.798	0.058	0.228	1.060	(0.678–1.656)	0.798

**Chronic diseases (ref. yes)**										
No	0.290	0.149	1.337	(0.999–1.790)	0.550	-0.107	0.178	0.899	(0.634–1.274)	0.550

**Smoking currently (ref. no)**										
Yes	0.087	0.135	1.091	(0.837–1.422)	0.684	0.067	0.164	1.069	(0.775–1.474)	0.684

**Physical activity level (ref. moderate/high)**										
Inactive	―	―	―	―	―	0.774	0.123	2.169	(1.704–2.762)	<0.001

**Sedentary behavior (ref. no)**										
Yes	0.763	0.123	2.145	(1.685–2.730)	<0.001	―	―	―	―	―

B=Regression coefficient; S.E. = Standard Error; Exp(B) = Odds Ratio (OR); CI = Confidence Interval.

a All variables except ‘Physical activity level’ were included as co-variates in multivariate analysis.

b All variables except ‘Sedentary behavior’ were included as co-variates in multivariate analysis.

## 4 Discussion

To our knowledge, this is the first study that investigated physical activity patterns and sedentary behaviors among Bangladeshi people during the COVID-19 pandemic, and provides a snapshot of such issues. This survey was conducted during a 10-day period, while the number of newly diagnosed cases was increasing in Bangladesh, and during which, substantial restrictions that included spatial distancing, home quarantine, social isolation, and travel restriction were in place. In a prior study focused on mental health conducted earlier during the outbreak in Bangladesh, we reported that 55.3% participants did not engage in physical exercise while in home quarantine, and 33.9% browsed internet more than 6 h per day [39]. We also reported that those individuals who reported vulnerable mental states (i.e., depression, anxiety, and stress) were significantly more likely not to engage in physical exercise and to browse the internet for longer periods of time.

Physical inactivity, a major risk factor for global mortality, accounts for 3.2 million deaths each year worldwide [40]. Not getting enough physical activity, including among those individuals who have no other associated risk factors, can lead to an increased risk of heart disease. Physical inactivity can also increase the likelihood of other risk factors for developing heart disease, such as obesity, high blood pressure, high blood cholesterol levels, and type 2 diabetes [41]. The fear of being infected and the mobility restrictions imposed during the COVID-19 pandemic may dissuade people from attaining the recommended levels of physical activity. In the present study, we found that nearly 38% of participants were physically inactive during the COVID-19 pandemic. Direct comparisons with these findings are quite difficult due to the lack of studies employing a similar instrument in Bangladesh. Here, we found that the prevalence of physical inactivity was significantly higher among young people (42.5%) compared to those individuals older than 25 years of age (27.7%). Young people spend more time on electronic devices than other age groups [42]. While confined at home because of COVID-19, young people could spend more time on electronic devices, leading them to increase the time spent as physically inactive. As corroboration of such assumption, single individuals (likely younger) were more likely to be physically inactive.

People with higher education levels were more inclined to report physical inactivity. This finding is in conflict with previous research studies that indicated that higher education levels are associated with higher degrees of involvement in physical activity [43, 44]. The discrepancies in this finding may be due to the situation imposed by COVID-19. Indeed, the prevalence of physical inactivity was significantly higher among students (46%) compared to all other groups, and was anticipated, considering the promotion of online activities during the COVID-19 pandemic. Since all the educational institutions were closed, students would be more prone to screen exposure in the context of both social media interactions, games or even studies online, and these trends may facilitate the emergence of mental and behavioral stress. People who were living in urban settings reported higher prevalence of physical inactivity. Participation in physical activity is largely determined by physical and social environmental factors that influence access, availability, and utilization [45, 46]. In Bangladesh, divisional cities are densely populated, and the number of COVID-19 cases was comparatively large. Accordingly, main factors favoring physical inactivity included being fearful of exposure to COVID-19, closed sports facilities, unavailability of friends to exercise with, and a lack of interest in pursuing physical activities during the COVID-19 pandemic [47].

The findings of this study also indicate that nearly 21% participants had high sedentary behavior (i.e., time spent on sedentary activities >8hours/day) during the COVID-19 pandemic. Prolonged sedentary behavior induces adaptations that negatively decondition cardiorespiratory fitness and metabolic profiles, and are therefore intimately related to disease prevention [48, 49]. It is possible that insufficient participation in physical activity over extended periods during the COVID-19 emergency may turn into sedentary behaviors. Females had higher prevalence of sedentary behaviors compared to males, similar to previous reports, possibly reflecting additional cultural and social norms [50]. Additional factors for high sedentary behaviors included being young, being a student, being from an upper-class family, and living in urban settings. Since these factors were also associated with physical inactivity, it is not surprising that they also contributed to increased sedentary behaviors.

The unique importance of physical activity and of restricting sedentary behaviors cannot be overstated, considering their beneficial effects on health in general, and also on specific elements related to the COVID-19 pandemic, such as modulation of the immune system [51]. Therefore, the WHO and many other professional societies recommend the adoption of specific exercise programs and daily strategies including home-based exercise programs to maintain a physically active lifestyle during the pandemic [52, 53].

### 4.1 Limitations

This study has some limitations that must be considered when interpreting the results. The present research adopted an online self-report methodology that may be susceptible to potential biases (e.g., social desirability and memory recall). In addition, the study was cross-sectional in nature and therefore we cannot infer causality between any of the variables examined. Furthermore, due to the online survey and convenience sampling technique, participants were predominantly educated young adults and students, which might affect the generalizability of the findings.

## 5 Conclusions

Physical inactivity is prevalent among the Bangladeshi population during the COVID-19 pandemic, and appears to be largely impacted by socio-demographic factors. Moreover, one-fifth of the cohort reported high sedentary behaviors. The findings suggest that there is a need to promote regular physical exercise in the context of home quarantine measures and increase awareness to induce cogent avoidance of activities related to sedentary behaviors during the COVID-19 outbreak.

## Declarations

### Author contribution statement

M. Rahman: Conceived and designed the experiments; Performed the experiments; Contributed reagents, materials, analysis tools or data; Wrote the paper.

M. Islam: Conceived and designed the experiments; Performed the experiments; Analyzed and interpreted the data; Contributed reagents, materials, analysis tools or data; Wrote the paper.

M. Bishwas and David Gozal: Contributed reagents, materials, analysis tools or data; Wrote the paper.

M. Moonajilin: Conceived and designed the experiments; Wrote the paper.

### Funding statement

This research did not receive any specific grant from funding agencies in the public, commercial, or not-for-profit sectors.

### Competing interest statement

The authors declare no conflict of interest.

### Additional information

No additional information is available for this paper.
